# Advancing Protein Structure and Ligand Binding Studies with Quantum Crystallography

**DOI:** 10.1063/4.0000876

**Published:** 2025-10-27

**Authors:** Maura Malinska

**Affiliations:** University of Warsaw, Warsaw, mazowieckie, Poland

## Abstract

Molecular recognition in biological systems is governed by non-covalent interactions that drive protein-ligand binding, enzyme catalysis, and drug action. Quantum crystallography provides an advanced framework for understanding these interactions by integrating high-resolution X-ray diffraction data with charge density reconstruction techniques. This approach enables a detailed analysis of electrostatic interactions, which are critical for ligand specificity and binding affinity.

In this talk, we explore how quantum crystallography tools enhance our understanding of protein-ligand interactions across different biological targets. The aspherical atom databank approach and electrostatic energy analysis have been successfully applied to systems such as androgen receptor inhibitors (e.g., bicalutamide)^1^, protein kinases targeted by sunitinib^2^, vitamin D receptor agonists^3^, and nonsteroidal anti-inflammatory drugs (NSAIDs) interacting with cyclooxygenases (COX-1 and COX-2)^4^. Our findings demonstrate that electrostatic complementarity plays a fundamental role in ligand recognition, dictating both binding strength and selectivity.

Beyond ligand interactions, quantum crystallography also provides a powerful tool for refining protein structures.^5^ The transferable aspherical atom model (TAAM) refinement improves electron-density maps, enhances the visibility of hydrogen atoms, and allows for more accurate modeling of protein and nucleic acid structures at ultrahigh resolution. This method lowers conventional refinement R factors and improves atomic displacement parameters, making it an essential approach for studying biomolecular interactions at an unprecedented level of detail.

This presentation will highlight the application of quantum crystallography in both protein structure refinement and drug discovery, demonstrating how charge density methods can refine protein-ligand interaction models and guide the rational design of therapeutics.
